# DNA-PKcs controls calcineurin mediated IL-2 production in T lymphocytes

**DOI:** 10.1371/journal.pone.0181608

**Published:** 2017-07-27

**Authors:** Ara Kim Wiese, Marie Schluterman Burdine, Richard H. Turnage, Alan J. Tackett, Lyle J. Burdine

**Affiliations:** 1 Department of Biochemistry and Molecular Biology, University of Arkansas for Medical Sciences, Little Rock, Arkansas, United States of America; 2 Division of Surgical Research, University of Arkansas for Medical Sciences, Little Rock, Arkansas, United States of America; 3 Department of Surgery, University of Arkansas for Medical Sciences, Little Rock, Arkansas, United States of America; 4 Department of Transplant Surgery, University of California San Francisco, San Francisco, California, United States of America; Univerzitet u Beogradu, SERBIA

## Abstract

Loss of DNA-dependent protein kinase catalytic subunit (DNA-PKcs) activity in mammals results in severe combined immuno-deficiency (SCID). This SCID phenotype has been postulated to be due solely to the function of DNA-PKcs in V(D)J recombination, a process critical for lymphocyte maturation. However; we show that DNA-PKcs is required for IL-2 production via regulation of the calcineurin signaling pathway. Reducing DNA-PKcs activity in activated T cells either by shRNA or an inhibitor significantly reduced IL-2 production by blocking calcineurin activity and the translocation of NFAT into the nucleus. Additionally, we show that DNA-PKcs exerts its effect on calcineurin by altering the expression of the endogenous calcineurin inhibitor Cabin1 through activation of the kinase CHK2, a known Cabin1 regulator. The discovery of DNA-PKcs as a potent regulator of IL-2 production will drive continued investigation of small molecule inhibition of this enzyme within the clinic.

## Introduction

The catalytic subunit of DNA-dependent protein kinase (DNA-PKcs) is a 460 kDa polypeptide member of the PI3k family. It was initially discovered to be a key component in non-homologous end-joining (NHEJ) which is the predominant pathway used to repair DNA double strand breaks in mammalian cells and is critical for V(D)J recombination [1, 2]. DNA-PKcs is believed to serve as a recruiting and scaffolding protein for DNA ligase [3]. Knock out of DNA-PKcs activity in mammals results in a Severe Combined Immunodeficiency (SCID) phenotype which is characterized by diminished levels of mature B and T cells [4–6]. This has been attributed to disruption of V(D)J recombination which is necessary for lymphocyte development and responsible for both antibody and T cell receptor diversity [7, 8]. Given this profound effect, we suspected that this enzyme is involved in other aspects of the immune response including Interleukin-2 (IL-2) signaling since the disruption of the IL-2 pathway in IL-2 receptor mutants also results in a SCID phenotype [9]. Of note, DNA-PKcs has previously been associated with multiple receptor signaling pathways including EGF, RET, and the insulin signaling pathway and phosphorylates key molecules associated with cell growth, e.g. AKT [10–14].

Well described, IL-2 is a T cell-derived cytokine that influences a multitude of key elements in the immune response including the proliferation and differentiation of B and T lymphocytes [15]. Expression of IL-2 is initiated upon calcineurin activation. Calcineurin is a calcium and calmodulin-dependent protein serine/threonine phosphatase that upon activation, dephosphorylates Nuclear Factor of Activated T-cells (NFAT) allowing it to translocate to the nucleus and upregulate expression of target genes (including IL-2) [15–17]. IL-2 then binds to its receptor IL-2R, expressed on the surface of lymphocytes, to induce signaling that impacts both arms of the immune response, humoral and cellular immunity [18]. IL-2 is known to promote the expansion and maturation of B and T lymphocytes and regulates the differentiation of T cells into effector or regulatory T cells [15–17].

To evaluate the function of DNA-PKcs in this pathway, we inhibited its activity by either shRNA or the commercially available inhibitor NU7441 in Jurkat cells, a human T cell line, and analyzed the effect on IL-2 levels. Inhibiting DNA-PKcs in activated Jurkat cells resulted in reduced calcineurin activity, loss of NFAT translocation to the nucleus and decreased IL-2 expression. We showed that this effect was linked to the calcineurin inhibitor, Cabin1. Cabin1 directly binds to activated calcineurin and blocks its dephosphorylation of NFAT. Overexpressing full length Cabin1 or its N-terminal region in Jurkat cells has been shown to reduce IL-2 expression by inhibiting the calcineurin-NFAT pathway [19, 20]. Cabin1 was also identified to function in DNA damage by inhibiting activity of p53 [21]. Through these studies it was revealed that phosphorylation of Cabin1 by the checkpoint kinase CHK2 targets it for ubiquination and degradation [22]. Interestingly, phosphorylation by DNA-PKcs is known to regulate activity of CHK2. DNA-PKcs phosphorylates CHK2 at site Thr68 thereby activating the kinase [23]. We showed that inhibiting DNA-PKcs in Jurkat cells resulted in a decrease in CHK2 phosphorylation causing an increase in Cabin1 expression. This novel pathway for regulation of IL-2 signaling indicates a much broader function for DNA-PKcs in the immune system than previously understood and further explains the development of a SCID phenotype in mice lacking DNA-PKcs activity.

## Materials and methods

### Materials

PHA-L, PMA, X-treme GENE transfection reagent, and 0.1% poly-lysine solution were purchased from Sigma-Aldrich (St. Louis, MO). NU7441 was purchased from Selleckchem (Houston, TX). shRNA against DNA-PKcs was purchased from Origene (Rockville, MD). Dynabeads Human T-Activator CD3/CD28 was purchased from Thermo Fisherscientific (Waltham, MA).

### Cell culture

Human peripheral blood mononuclear cells (PBMC,) and Jurkat cells were purchased from ATCC (PCS-800-011, Manassas, VA). Cells were maintained at 37°C in a humidified atmosphere composed of 5%CO_2_. Jurkat cells were cultured in RPMI 1640 medium which was supplemented with 10%FCS and human PBMC was cultured in RPMI 1640 medium which was supplemented with 10% FCS and pen/strep. Both Jurkat cells and PBMC were stimulated with PHA (50ng/mL) and PMA (1μg/mL) for 24 hours prior to harvesting for IL-2 detection or 6 hours prior for western blot analysis. The NU7441 DNA-PKcs inhibitor was added at varying concentrations at the time of stimulation.

### Knockdown of DNA-PKcs in Jurkat cells

Jurkat cells (5 x 10^5^ cells/well) were grown in 6-well plates. The cells were transfected with short-hairpin RNA (shRNA) plasmids generated by Origene. (2.5 μg of scramble or 2.5 and 5 μg of specific to DNA-PKcs) using X-treme GENE and incubated for 72 hours. Four shRNA plasmids were obtained from Origene that target various regions of DNA-PKcs. The shRNA plasmid that provided the best knock down of DNA-PKcs expression was used for our experiments. The cells were subjected to Western blot analysis and IL-2 ELISA assay. shRNA plasmids against DNA-PKcs was purchased from Origene (Rockville, MD).

### Cell lysis and nuclear extract

Cells were washed with cold PBS twice and centrifuged at 5000rpm for 5 min. For the nuclear extract, pellets were suspended with 800μL of 10mN HEPES lysis buffer (10mM HEPES at pH 7.9, 10mM KCl, 1mM DTT, and 1X protease and phosphatase inhibitor) and incubated on ice for 15min. 50uL of NP-40 (10% in water) was added and the pellets were mixed for 10 sec. Lysates were centrifuged for 5 min at 4°C at 13,000rpm. The supernatant solution which is containing the cytosolic fraction was discarded and the pellets were resuspended in 20mM HEPES lysis buffer (20mM HEPES at pH 7.9, 0.4M NaCl, 1mM DTT, and 1X protease and phosphatase inhibitor) and incubated on ice for 15min with intermittent mixing. Lysates were centrifuged for 10 min at 4°C at 13,000rpm. The supernatant containing nuclear extract was stored at -20°C until they were used for the Western blot analysis. For total cell lysates, cell pellets were resuspended with 100uL of RIPA buffer (150mM NaCl, 1% Triton X-100, 0.1% SDS, and 50mM Tris at pH 8.0) and incubated on ice for 10min. The lysates were centrifuged for 10min at 4°C at 13,000rpm and the supernatant solutions were stored at -20°C until they were used for the Western blot analysis.

### Western blot analysis

Nuclear extract lysates were separated on 3–8% Tris-Acetate gels (invitrogen). Total cell lysates were separated on 4–20% Tris-Glycine gels (Bio-Rad). Gels were transferred onto PVDF membrane (Millipore) for 2h in the cold room at 100V. Immunoblotting was performed using following antibodies: pDNA-PKcs at S2056 (ab18192, Abcam), DNAPK (ab53701, Abcam), pNFAT2 at S237 (ab183023, Abcam), CABIN1 (12660S, Cell signaling), GAPDH (MAB374, Millipore), and Lamin B1 (ab16048, Abcam). HRP-conjugated secondary antibody anti-Rabbit and anti-Mouse (7074S and 7076S, Cell Signaling) was used.

### Cell viability assay

Cell viability assay was performed using Promega CellTiter 96 AQueous One Solution Cell Proliferation Assay (Madison, WI) and following the manufacturer’s protocol. Briefly, 100uL of PBMC or Jurkat cells were plated in a 96-well plate and treated with various concentration of NU7441 for 48h. CellTiter solution (20uL/100uL of cell suspension) was added to the cells and the plate was incubated for 3h at 37°C and the absorbance at 490nm was recorded using SynergyHTX (BioTek, Winooski, VT) plate reader.

### Immunofluorescence of NFAT

Jurkat cells were plated in poly-lysine treated 35mm dish with glass-bottom (BioTek) overnight. The cells were fixed with 4% formaldehyde and permeabilized with permeabilization solution (0.2% Triton X-100 + OVA solution (0.1 mg/ml), 0.01% sodium azide). NFAT was probed with anti-NFAT2 (ab2796, Abcam) in permeabilization solution overnight at 4°C, and then washed with PBS. Fluorescein (FITC)-conjugated AffiniPure Donkey Anti-Mouse IgG (715-095-151, Jackson ImmunoResearch) was applied to the cells for 1h at RT. The cells were washed with PBS and ProLong Antifade reagent with DAPI (Molecular Probes) was applied. All samples were analyzed on an Olympus Fluoview FV1000 laser confocal microscope. Images from all microscopy experiments were processed using the FV10-ASW 3.1 Viewer (Olympus).

### Detection of secreted IL-2

Secreted IL-2 was detected by Human IL-2 ELISA Kit from Thermo Scientific (Waltham, MA). The manufacturer’s protocol was followed. Prior to harvesting, cells were treated with PHA (50ng/mL) and PMA (1μg/mL) for 24 hours with or without the NU7441 inhibitor. Jurkat cells stimulated with the anti-CD28/CD3 dynabeads were done so according to the manufacturer’s protocol at a 1:1 ratio for 24 hours prior to harvesting. After stimulation, supernatant samples of Jurkat cells or PBMCs (2 million cells/mL) were collected and diluted 10 times before the assay. IL-2 standards and samples (50 μL) and Biotinylated antibody reagents (50 μL) were added to each well and the plate was incubated for 3h at RT. The plate was washed 3 times and 100 μL of Streptavidin-HRP solution was added. After 30 min of incubation at RT, the plate was washed 3 times. TMB substrate (100 μL) was added and incubated for 30min in the dark at RT. Stop solution was added and the absorbance of each well was read at 450nm using the plate reader.

### Detection of calcineurin and mTOR activities

Calcineurin phosphatase activity was detected by Calcineurin Cellular Activity Assay Kit from Millipore (Billerica, MA) and mTOR signaling was detected by Calcium Detection Kit from Abcam (Cambridge, MA). The manufacturer’s protocols were followed. Briefly, Jurkat cells (2 million cells/mL) were lysed using lysis buffer. For calcineurin activity, the cell lysates were desalted by gel filtration to remove free phosphates before the assay and were subjected to calcineurin activity assay. The absorbance of each sample was read at 620nm using the plate reader. For mTOR ELISA assay, the cell lysates were added to each well and antibody against phosphorylated mTOR at serine 2448 was used to detect the activity of mTOR signaling. The absorbance of each sample was read at 450nm using the plate reader.

### Measurements of calcium ions

Intracellular concentration of calcium ions was measured by Calcium Detection Assay Kit from Abcam (Cambridge, MA). The manufacturer’s protocols were followed. Briefly, Jurkat cells (2 million cells/mL) were lysed using cold PBS with 0.1% NP-40. The cell lysates were diluted 10 times before use. After following the assay protocol, the absorbance of each sample was read at 575nm using the plate reader.

### Statistical analysis

Assays to monitor IL-2 levels, calcineurin and mTOR activities, and Ca^2+^ ion levels were performed in both technical triplicate and biological triplicate. Standard student t-test were performed to compare group means. Means with p-value below 0.05 were considered statistically different.

## Results

### DNA-PKcs regulates IL-2 secretion in T cells

**Fig 1:**
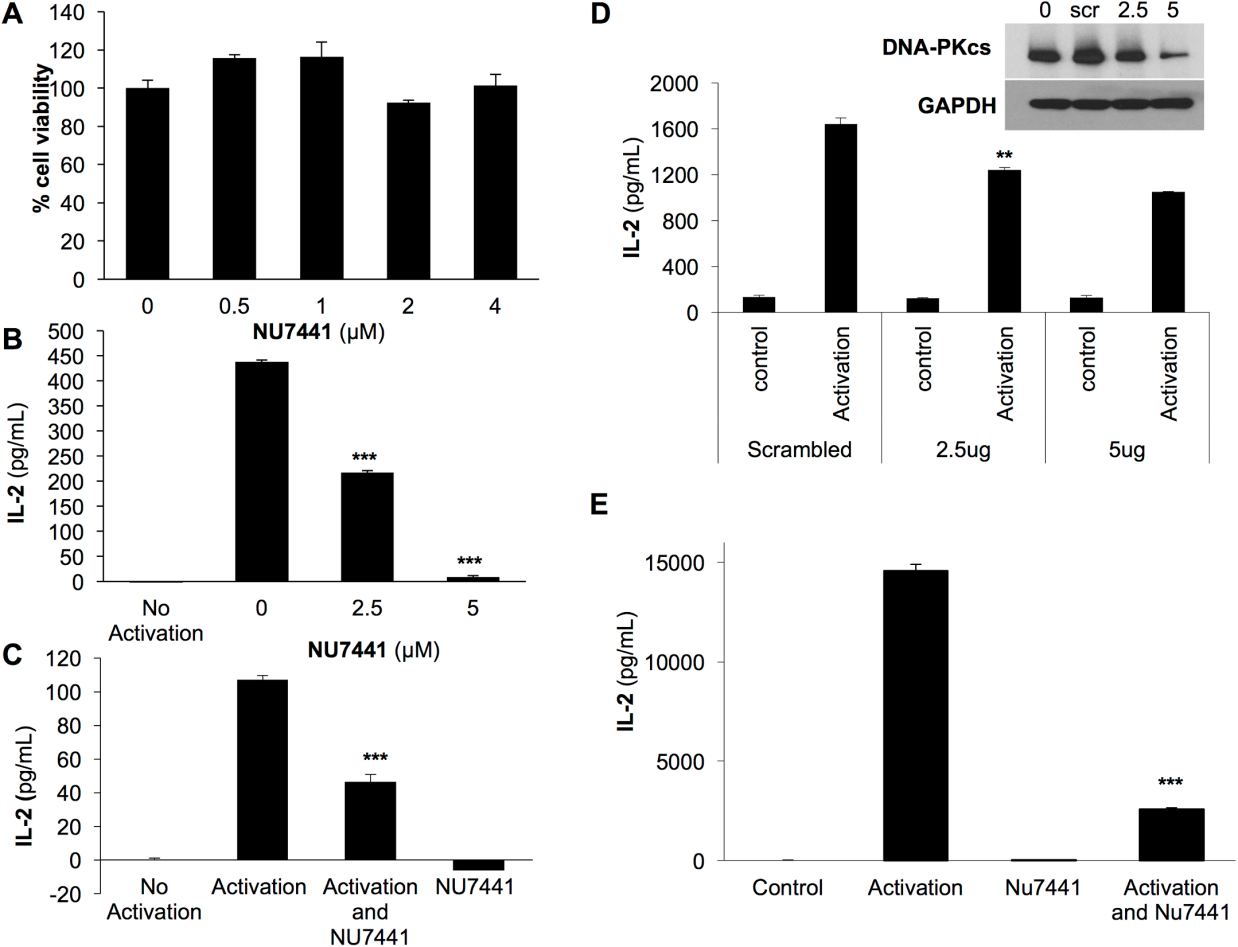
Inhibition of DNA-PKcs in T cells and PBMCs blocks IL-2 production. A) Jurkat cells were treated with the DNA-PKcs inhibitor NU7441 at varying concentrations for 48 hours and no significant reduction in viability was detected. B) Jurkat cells were stimulated with PMA (50 ng/mL)+PHA (1 μg/mL), treated with NU7441, and analyzed for IL-2 production 24 hours later. NU7441 treatment significantly blocked IL-2 secretion. C) IL-2 production stimulated by activation of Jurkat cells with anti-CD28/CD3 dynabeads at a 1:1 ratio for 24 hours was inhibited by NU7441 treatment. D) Treatment of Jurkat cells with shRNA reduced DNA-PKcs expression at 2.5 and 5 μg as seen by western blot analysis. Loss of DNA-PKcs expression significantly reduced IL-2 production. E) NU7441 significantly reduce IL-2 production following activation with PHA+PMA in PBMCs. ** p< 0.002 *** p<0.001 error bars = s.d.

The immune cytokine IL-2 is a key element of the immune response affecting both the humoral and cell-mediated arms of the immune system. To determine if DNA-PKcs regulates T cell-mediated IL-2 production, we evaluated the effect of the DNA-PKcs inhibitor NU7441 on IL-2 in Jurkat cells. NU7441 is a potent and specific inhibitor of DNA-PKcs which does not interfere with ATR or ATM activation [24]. We first determined that NU7441 at varying concentrations did not alter the viability of Jurkat cells (Fig 1A). Next, we monitored the production of IL-2 in Jurkat cells treated with NU7441. In Fig 1B, we observed the expected spike in IL-2 levels following 24 hour stimulation with PMA+PHA. In the presence of the inhibitor, the level of IL-2 was significantly decreased with 2.5 μM of NU7441 and further decreased with 5 μM (Fig 1B). During an immune response, T cells are typically stimulated by activation of the T cell receptor (TCR). To determine if DNA-PKcs was acting in a TCR directed pathway, we repeated the IL-2 production assay after stimulating T cells with anti-CD28/CD3 dynabeads which activate the TCR. Cells were harvested 24 hours after stimulation and treatment with or without the NU7441 inhibitor. As seen with PMA+PHA activation, NU7441 significantly blocked IL-2 production stimulated by anti-CD28/CD3 dynabeads (Fig 1C). To confirm that the effect of IL-2 secretion was specific to DNA-PKcs and not a side effect of the inhibitor, we knocked down expression of DNA-PKcs using short hairpin RNA plasmids (shRNA). The protein level of DNA-PKcs was reduced with shRNA indicating that the knock down of DNA-PKcs was successful (Fig 1D). Loss of DNA-PKcs expression significantly inhibited secretion of IL-2 in T cells following activation with PMA+PHA confirming DNA-PKcs as a critical regulator of IL-2 production (Fig 1D).

We next wanted to solidify our findings by examining the effect of DNA-PKcs inhibition on IL-2 production in more clinically relevant human primary immune cells. Therefore; we inhibited DNA-PKcs activity in Peripheral Blood Mononuclear Cells, PMBC, and evaluated IL-2 production. Like Jurkat cells, NU7441 did not affect cellular viability but did significantly reduce the level of IL-2 produced following activation with PMA+PHA (S1 Fig and Fig 1E).

### DNA-PKcs inhibition blocks nuclear localization of NFAT

**Fig 2:**
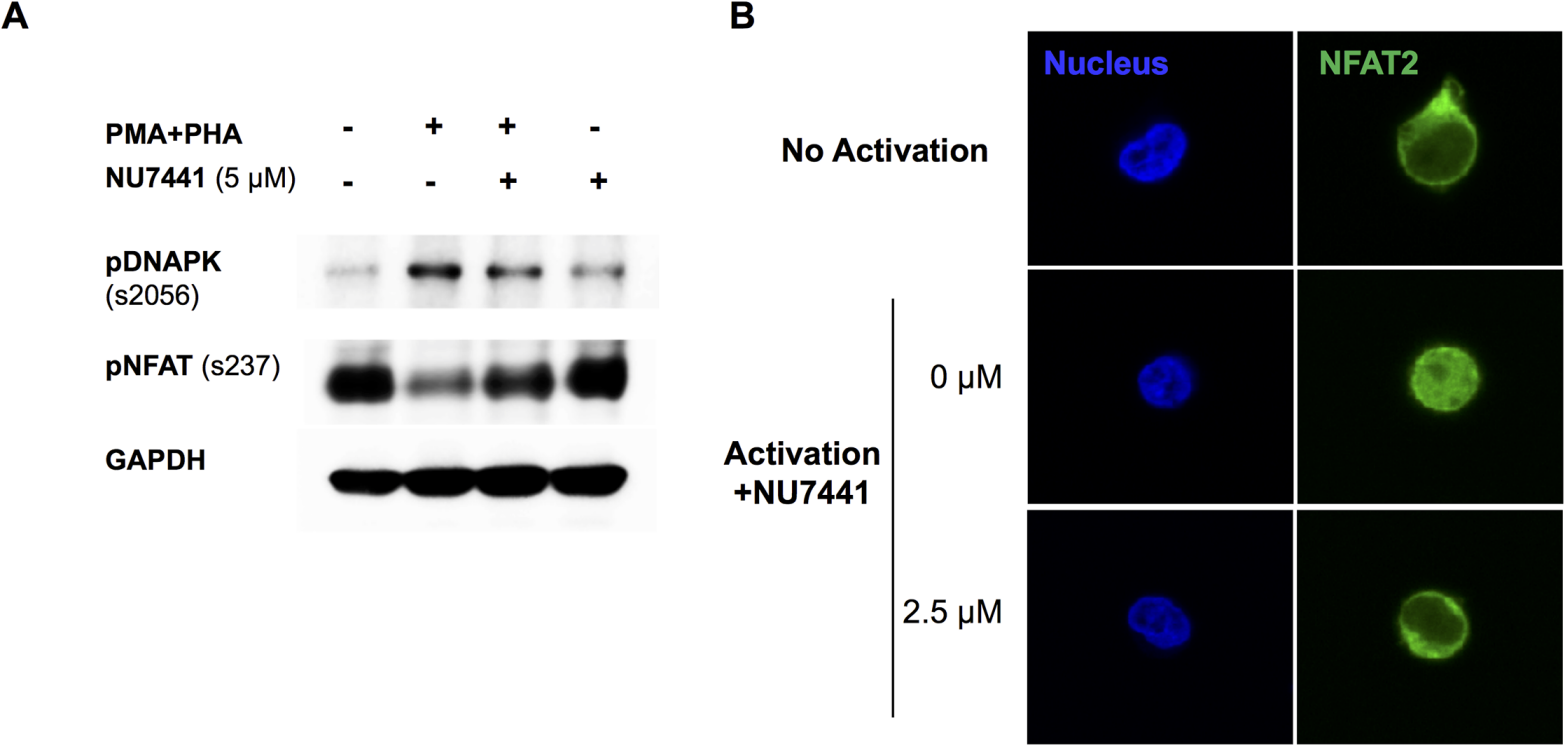
Inhibition of DNA-PKcs blocks translocation of NFAT to the nucleus. A) Western blot analysis of Jurkat cell lysates showed activation of T cells with PMA+PHA induced phosphorylation of DNA-PKcs at site s2056 (pDNA-PK) and dephosphorylated NFAT at s237 (pNFAT). Treatment with NU7441 inhibited the dephosphorylation of NFAT at site s237 which is critical for its translocation to the nucleus. GAPDH was used as a loading control. B) Immunocytochemistry analysis of Jurkat cells treated with NU7441. The inhibitor (2.5 μM) blocked translocation of NFAT to the nucleus following activation with PMA+PHA. Nuclei were stained with Dapi. 40X images are shown.

IL-2 production is initiated by dephosphorylation and translocation of the transcription factor NFAT to the nucleus. Therefore; we examined the effect of DNA-PKcs inhibition on NFAT in Jurkat cells by western blot and immunocytochemistry. In Fig 2A, we show that activation of Jurkat cells with PMA+PHA induced phosphorylation of DNA-PKcs at serine 2056, an activation site [25]. Additionally, NU7441 effectively inhibited DNA-PKcs phosphorylation confirming that NU7441 successfully inhibits DNA-PKcs activity. Without activation, NFAT was phosphorylated (s237) and resided in the cytoplasm in Jurkat cells (Fig 2A and 2B). Upon activation, NFAT was dephosphorylated and translocated to the nucleus. However; in the presence of NU7441, NFAT remained phosphorylated and nuclear localization was prevented, further suggesting that DNA-PKcs is critical for proper T cell signaling (Fig 2A and 2B).

### DNA-PKcs inhibition reduces calcineurin activity in T cells

**Fig 3:**
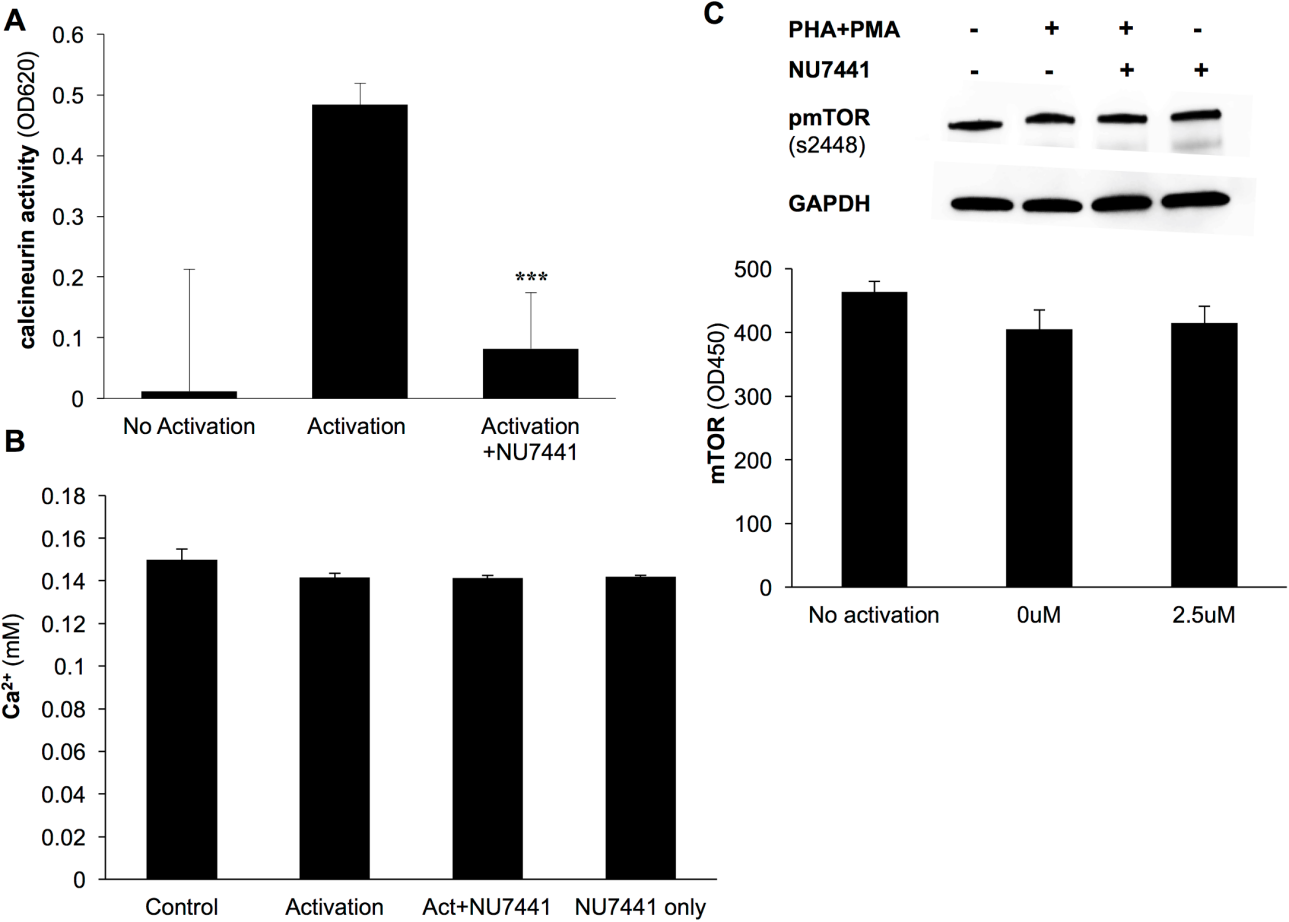
DNA-PKcs inhibition blocks calcineurin activity in T cells. A) Jurkat cells were activated with PMA+PHA, treated with the DNA-PKcs inhibitor NU7441 (2.5μM) and monitored for calcineurin phosphatase activity. Inhibition caused a significant reduction in calcineurin activity. B) Level of Ca^2+^ in Jurkat cell lysates following activation with PMA+PHA was monitored. Ca^2+^ levels were not affected by the addition of the NU7441 inhibitor. C) Western blot and Elisa analysis of active phosphorylated mTOR in activated Jurkat cells indicated that inhibition of DNA-PKcs does not alter mTOR activation. ***p<0.001 error bars = s.d.

As mentioned above, the regulation of NFAT is mediated via phosphorylation. During T cell activation, calcineurin, a calcium/calmodulin-dependent serine-threonine phosphatase, is activated and dephosphorylates NFAT allowing it to translocate to the nucleus to initiate transcription. Therefore, we evaluated the effect of DNA-PKcs on calcineurin activity in Jurkat cells. The phosphatase activity of calcineurin was greatly increased in the presence of PMA+PHA, however the activity was significantly inhibited with NU7441 treatment (Fig 3A). Since the activity of calcineurin is regulated by the intracellular Ca^2+^ ion concentration, the level of calcium ions was monitored. In the presence of NU7441 following activation, there was no change in the concentration of Ca^2+^ ions (Fig 3B) proving that DNA-PKcs does not regulate calcineurin activity by altering the influx of Ca^2+^ but by a different unknown mechanism.

Mammalian target of rapamycin, mTOR, a member of the PI3K kinase family, is a second signaling pathway initiated following T cell activation [26]. Therefore; we evaluated the effect of DNA-PKcs on the mTOR pathway. Activated Jurkat cells with or without NU7441 treatment were subjected to an mTOR assay which detects the level of activated mTOR with an antibody specific to phosphorylated ser2448. Results from the assay along with western blot analysis of phosphorylated mTOR showed that loss of DNA-PKcs activity did not alter mTOR activation in T cells. (Fig 3C). This further indicates a function for DNA-PKcs in T cells that is specific to the calcineurin signaling pathway.

### DNA-PKcs regulates expression of the calcineurin inhibitor Cabin1

**Fig 4:**
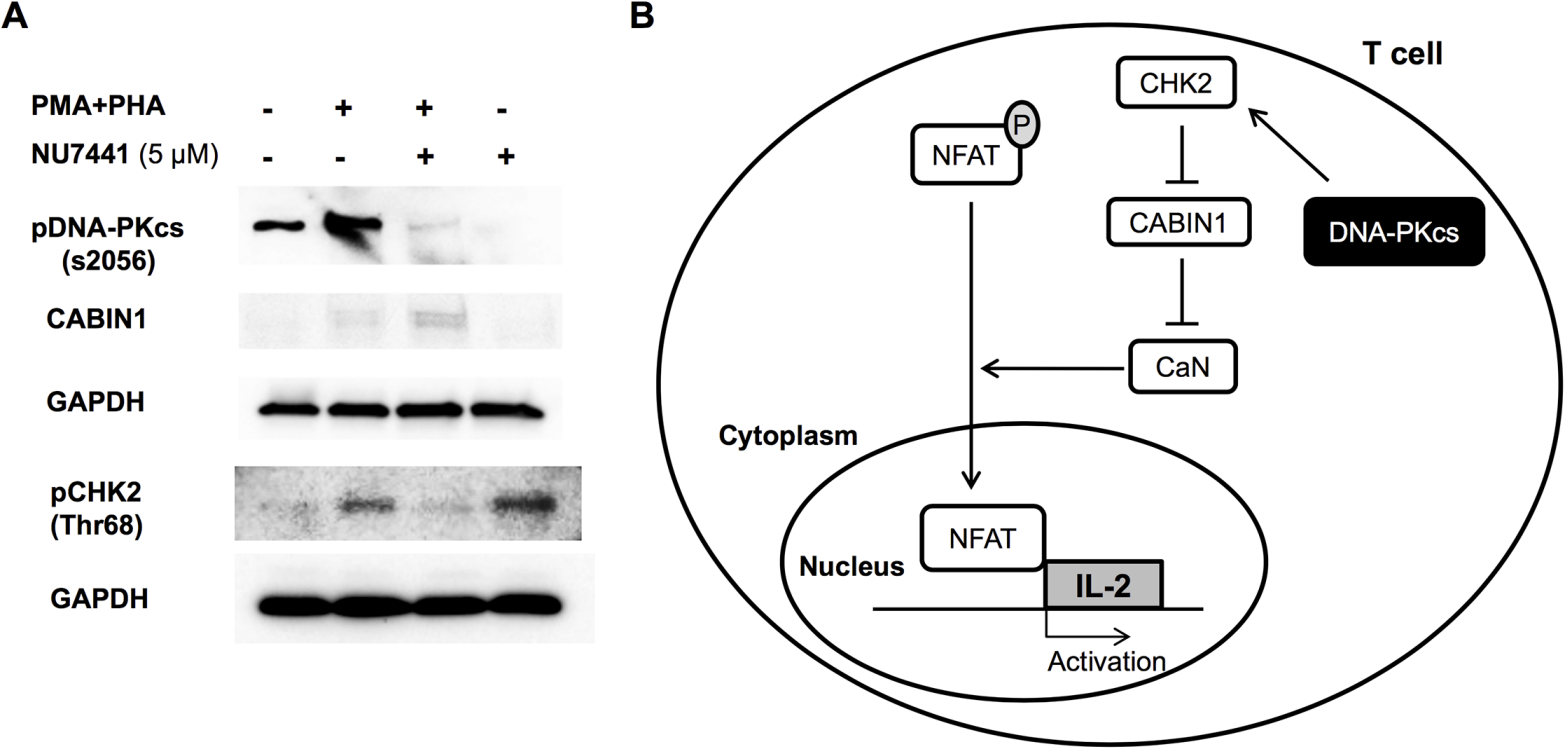
Inhibition of DNA-PKcs reduces phosphorylation of CHK2 and stabilizes the calcineurin inhibitor, Cabin1. A) Western blot analysis of Jurkat lysates following activation with PMA+PHA and NU7441 treatment. Activation increased phosphorylation of DNA-PKcs and CHK2. DNA-PKcs inhibition reduced CHK2 phosphorylation and elevated Cabin1 expression. GAPDH was used as a loading control. B) Schematic depicting the signaling pathway in T cells used by DNA-PKcs to regulate IL-2 production. DNA-PKcs phosphorylates CHK2 which in turns phosphorylates Cabin1 targeting it for destruction. This alleviates calcineurin inhibition causing an increase in translocation of NFAT and IL-2 production. CaN, calcineurin.

The endogenous calcineurin inhibitor, Cabin1, binds calcineurin preventing the dephosphorylation of NFAT and transcription of immune cytokines including IL-2 [19, 20]. Cabin1 works in a similar fashion in the DNA damage repair pathway by binding p53 preventing its interaction with DNA [21, 22]. In this pathway, Cabin1 expression is controlled by checkpoint kinase CHK2. DNA damage signals the phosphorylation of CHK2 by DNA-PKcs at site T68 which stimulates CHK2 to hyper-phosphorylate Cabin1 targeting it for ubiquitination and degradation [23]. In this study, we examined the relationship between DNA-PKcs, CHK2 and Cabin1 in the T cell signaling pathway. We show that following activation of T cells, phosphorylation of DNA-PKcs (s2056) is increased along with an increase in CHK2 phoshorylation at Thr68 (Fig 4A). Phosphorylation of both proteins was reduced with the NU744 inhibitor (Fig 4A) indicating that DNA-PKcs is partly responsible for CHK2 activation in T cells. However; inhibition of DNA-PKcs and subsequently activation of CHK2 caused an increase in Cabin1 expression (Fig 4A). This effect would result in a decrease in calcineurin activity and IL-2 production. These data highlight a novel mechanism by which DNA-PKcs regulates calcineurin signaling in T cells by its inhibitor Cabin1. A schematic of this mechanism is displayed in Fig 4B.

## Discussion

DNA-PKcs is a ubiquitously expressed enzyme with an increasing amount of functions defined in the literature. Not only is the enzyme critically important for NHEJ, but it has also been shown to phosphorylate a wide variety of substrates critical to cell growth, division, and homeostasis [10–14]. Mutations of DNA-PKcs in mammals present clinically with a SCID phenotype that is indistinguishable from other genetic causes of SCID [27]. Given its emerging function as a key regulator for numerous signaling transduction pathways, we hypothesized that DNA-PKcs not only affects the immune response through its role in V(D)J recombination but also by regulation of the calcineurin signaling pathway which stimulates the production of IL-2, a critical immune cell cytokine. Interestingly, DNA-PKcs has been previously reported to associate with proteins that bind to the antigen receptor response element in the IL-2 promoter region further suggesting a role for this protein in IL-2 regulation [28]. The IL-2 pathway has been extensively researched and has significant clinical importance particularly with respect to transplant, cancer, and cardiovascular biology. DNA-PKcs has not previously been linked to either mature T cell activation or the calcineurin signaling pathway. Using a Jurkat T cell model, we identified a novel mechanism where DNA-PKcs regulates T cell-mediated signaling by altering the expression of the calcineurin inhibitor, Cabin1. The function of Cabin1 in T cell signaling has been well-characterized as a negative regulator of calcineurin activity [19, 20]. We show that through Cabin1, DNA-PKcs can exert control over the immune response. Like DNA-PKcs and CHK2, Cabin1 is involved in the DNA damage repair pathway. Cabin1 functions to inhibit the pathway by binding to p53 preventing its ability to bind DNA and promote transcription of DNA repair genes [21, 22]. Expression of Cabin1 is altered in response to DNA damage through activation of ATM and its target kinase, CHK2. Phosphorylation and activation of CHK2 result in degradation of Cabin1 freeing p53 to bind to DNA. DNA-PKcs has not been shown to effect Cabin1 expression, however; it does phosphorylate and activate CHK2 in response to DNA damage [23]. Therefore; we hypothesized that activation of DNA-PKcs following T cell activation could alter Cabin1 expression via activation of CHK2. Our results support this hypothesis. The mechanism by which DNA-PKcs gets activated upon T cell activation is still unclear. T cell activation induces a multitude of kinase signaling cascades, some of which are known to activate DNA-PKcs in response to DNA damage (ERK1/2 and AKT) [29–31]. Therefore; it is reasonable to assume that following T cell activation DNA-PKcs is getting activated through one of these signaling pathways.

The results presented here underscore an additional role of DNA-PKcs in the immune system. Small molecule inhibition of DNA-PKcs is currently in Phase I clinical trials for cancer therapy with the idea being that chemoresistance can be usurped via disruption of a DNA double strand break repair pathway [32] (Clinicaltrials.gov). Our results suggest that inhibition of this enzyme will likely have an immediate and profound effect on T-cell signaling in addition to its well-established role in V(D)J recombination. While the outcome of these clinical trials and the benefit of DNA-PKcs inhibitors as cancer therapy are still being evaluated, one could hypothesize the outcome. Loss of IL-2 expression due to these inhibitors could result in a reduced anti-oncogenic T cell response counteracting any positive effect from the inhibition of DNA damage repair. The effect of DNA-PKcs on IL-2 production must be considered when deciphering the outcome of these trials.

This work also highlights a novel use for DNA-PKcs inhibitors. Single drug small molecule inhibition of both cell mediated and humoral immunity is a goal of transplant pharmacology. Given this data, we feel that DNA-PKcs is a worthwhile target for immunosuppresion in the transplant population as both an induction agent and possible maintenance therapy. Results from this study warrant investigation into the immunosuppression benefit of DNA-PKcs inhibition in transplant recipients.

## Supporting information

S1 Fig NU7441 treatment does not affect viability of PBMC. PBMCs were treated with NU7441 (1.25 and 2.5 μM) for 48 hours and monitored for viability. Viability was not affected by NU7441 treatment. error bars = s.d.
(TIF)
