# Supplementary figures and images for: DNA-PKcs controls calcineurin mediated IL-2 production in T lymphocytes
Source: PLoS One. 2017 Jul 27;12(7):e0181608. doi: 10.1371/journal.pone.0181608 (PMC5531461; doi:10.1371/journal.pone.0181608)

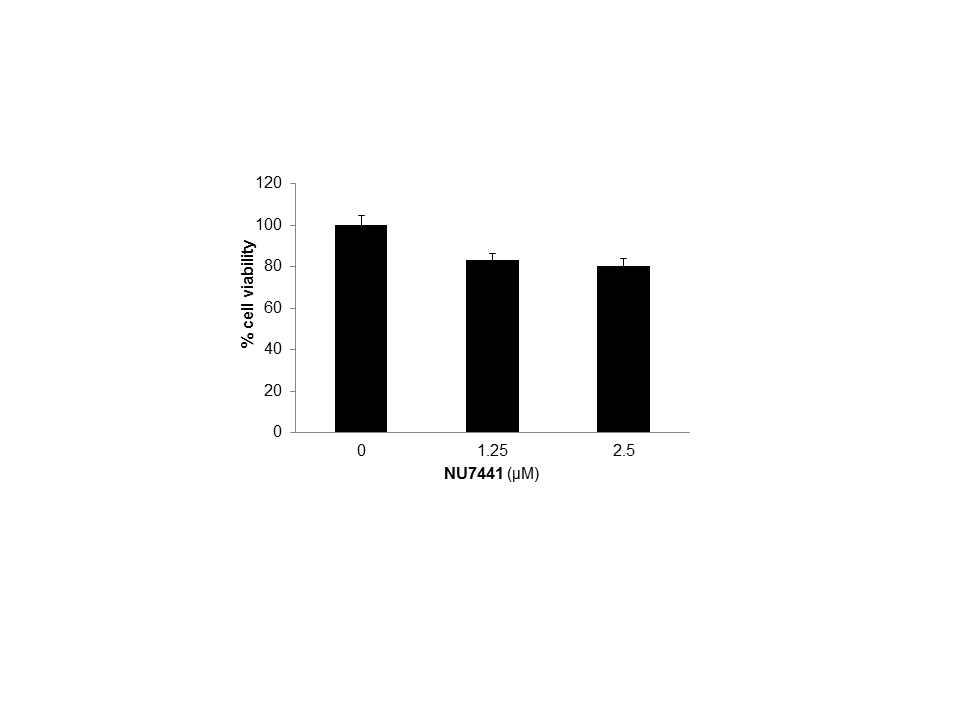

Supplement: S1 Fig — PBMCs were treated with NU7441 (1.25 and 2.5 μM) for 48 hours and monitored for viability. Viability was not affected by NU7441 treatment. error bars = s.d. (TIF) [file pone.0181608.s001.TIF]
